# Most Influential Qualities in Creating Satisfaction Among the Users of Health Information Systems: Study in Seven European Union Countries

**DOI:** 10.2196/11252

**Published:** 2018-11-30

**Authors:** Shahryar Eivazzadeh, Johan S Berglund, Tobias C Larsson, Markus Fiedler, Peter Anderberg

**Affiliations:** 1 Department of Health Science Blekinge Institute of Technology Karlskrona Sweden; 2 Department of Mechanical Engineering Blekinge Institute of Technology Karlskrona Sweden; 3 Department of Technology and Aesthetics Blekinge Institute of Technology Karlskrona Sweden

**Keywords:** health information systems, telemedicine, evaluation studies as topic, consumer behavior, treatment outcome, safety, efficiency, health care costs, ontology engineering, equation models

## Abstract

**Background:**

Several models suggest how the qualities of a product or service influence user satisfaction. Models such as the Customer Satisfaction Index (CSI), Technology Acceptance Model (TAM), and Delone and McLean Information Systems Success demonstrate those relations and have been used in the context of health information systems.

**Objective:**

This study aimed to investigate which qualities foster greater satisfaction among patient and professional users. In addition, we are interested in knowing to what extent improvement in those qualities can explain user satisfaction and whether this makes user satisfaction a proxy indicator of those qualities.

**Methods:**

The Unified eValuation using ONtology (UVON) method was used to construct an ontology of the required qualities for 7 electronic health (eHealth) apps being developed in the Future Internet Social and Technological Alignment Research (FI-STAR) project, a European Union (EU) project in electronic health (eHealth). The eHealth apps were deployed across 7 EU countries. The ontology included and unified the required qualities of those systems together with the aspects suggested by the Model for ASsessment of Telemedicine apps (MAST) evaluation framework. Moreover, 2 similar questionnaires for 87 patient users and 31 health professional users were elicited from the ontology. In the questionnaires, the user was asked if the system has improved the specified qualities and if the user was satisfied with the system. The results were analyzed using Kendall correlation coefficients matrices, incorporating the quality and satisfaction aspects. For the next step, 2 partial least squares structural equation modeling (PLS-SEM) path models were developed using the quality and satisfaction measure variables and the latent construct variables that were suggested by the UVON method.

**Results:**

Most of the quality aspects grouped by the UVON method are highly correlated. Strong correlations in each group suggest that the grouped qualities can be measures that reflect a latent quality construct. The PLS-SEM path analysis for the patients reveals that the effectiveness, safety, and efficiency of treatment provided by the system are the most influential qualities in achieving and predicting user satisfaction. For the professional users, effectiveness and affordability are the most influential. The parameters of the PLS-SEM that are calculated allow for the measurement of a user satisfaction index similar to CSI for similar health information systems.

**Conclusions:**

For both patients and professionals, the effectiveness of systems highly contributes to their satisfaction. Patients care about improvements in safety and efficiency, whereas professionals care about improvements in the affordability of treatments with health information systems. User satisfaction is reflected more in the users’ evaluation of system output and fulfillment of expectations but slightly less in how far the system is from ideal. Investigating satisfaction scores can be a simple and fast way to infer if the system has improved the abovementioned qualities in treatment and care.

## Introduction

### Background

The normative evaluation of health information systems is articulated through a frequently used set of keywords such as *acceptance* or *adoption* [1,2], *success* [3], and *satisfaction* [4,5]. Each of these keywords reminds us how a health information system inherits traits from its conceptual ancestors, that is, the information system, technology, and product. For an overall evaluation of these systems, one might measure how well these information systems succeed [6,7], how these technologies are accepted by users [8,9], or how the customers of these systems, patients, or professionals are satisfied with these products [10]. Below this layer of top indicators, there exist sets of constructs and relationships that cause success, acceptance, or satisfaction. Researchers have tried to capture and demonstrate through the models how success [6,10], acceptance [9], or satisfaction [11-13] are created by constructs such as perceived quality, perceived expectation, ease of use, and other variables. Some of these models have largely been employed in diverse contexts [14]. There are also models, whether novel or customized from the mainstream, that are specific to a smaller context such as health information systems [15-17].

The Customer Satisfaction Index (CSI) model family places the *satisfaction* construct at the core of their path structures. There, the satisfaction construct is affected by leading indicators such as perceived quality. At the same time, it impacts lagging indicators such as user loyalty. There are at least three versions of CSI widely being used. The original CSI model was introduced in Sweden [11]. The American Customer Satisfaction Index improved the Swedish version [12], and then the European Customer Satisfaction Index (ECSI) enhanced the American version [18]. The ECSI model consists of 9 latent construct variables [18], which are measured by a series of measure or manifest variables. Historically, CSI models have been used at macro levels where the satisfaction of customers at the national level or the level of an enterprise was the matter of concern. The wording of CSI models, such as the *customer* term, and the inclusion of some constructs, such as *loyalty*, suggest that measuring user satisfaction at the micro level, that is, the product level, was not their main target. However, the manifestation of the satisfaction concept in the CSI models through its 3 manifest variables [18] is versatile enough to measure satisfaction at both micro or macro levels with the same wording. CSI models introduce a way of measuring satisfaction scores through adding the weighted scores of those 3 variables, which inspires similar approaches in various disciplines. In comparison with using the CSI models for health information systems, one might consider the patient satisfaction models [19] that share a set of common constructs and relations with the CSI models but do not necessarily embed the same structures or components.

The Delone and McLean Information Systems Success (D&M IS) model, a prevalent model for analyzing the success of information systems, sets out the relationship between user satisfaction and quality dimensions [6,20]. In this model, 3 categories of qualities, information, system, and service contribute to user satisfaction. There are 2 other constructs, *net benefits* and *intention to use*, that are in a bidirectional impact relationship with *user satisfaction* [20]. Several studies have validated the causal relationships between the constructs in the D&M IS model [21]. In addition, there exists a long list of validated measures for each of the constructs [22]. The D&M IS model has broadly been used in the health information system context [23]. Furthermore, it has also been extended and customized to be more specific for this context, such as in the Human, Organization, and Technology Fit model [24], but the extension has been directed more toward a wider perspective of organization and technology. Although some studies have incorporated [25] or prioritized [26] more specific qualities, further investigation is needed to be more specific about the impacting qualities and their degree of importance.

Technology acceptance models, such as Technology Acceptance Model (TAM) as well as the Unified Theory of Acceptance and Use of Technology, which are supported by a great many of studies [8,9], placed the *acceptance* of a system or technology at the core of relationships. These models have been applied in health information system studies, although they have reported the significance of some of relationships differently [8]. The user acceptance of a health information system can be a prelude to or reflection of their satisfaction in using that system, but acceptance is not the same as satisfaction. Some researchers have considered *acceptance*, that is, the behavioral intention to use in TAM, an equivalent for *satisfaction* [27], but the *intention to use* is a different construct from *satisfaction* in a well-studied model such as D&M IS.

Contextualizing TAM by adding variables has been a common practice [8,28,29]. Indeed, contextualizing TAM for health information technology has led to the introduction of some frequently employed variables, such as *fit* [30]. However, there is a shortage of studies applying a systematic approach, such as *belief elicitation* [31], when introducing a new variable [8].

### Objectives

For the CSI, D&M IS, and TAM, the set of relationships between their proposed constructs have been already examined in various contexts [8,14,13]. Nevertheless, for a specific context such as health information systems, one might seek to develop new models, probably inspired by those that are well established, to expand a construct into more detailed constructs or find manifest variables more relevant to a case. For example, the constructs that represent the qualities of a system are generalized in those models or their variations as perceived quality [13]; system, service, or information quality [20]; and output quality [8]. However, none of those models represents a specific quality, such as safety, as a stand-alone construct.

In addition, finding a systematic approach to define manifests for construct variables, as mentioned before, is another direction for extending a model [8]. Many of the evaluation frameworks for health information systems suggest the qualities to evaluate [32], arranged as categories or domains. These frameworks implicitly suggest constructs and the manifests to each construct. Simultaneously, the end users of health information systems are another source for eliciting the qualities and their groupings [33].

In the forthcoming sections, we put forth a list of qualities that create and predict user satisfaction with health information systems. The qualities are embedded within a path model that demonstrates their relationships with user satisfaction. This study’s methods and materials are discussed in the Methods section. The qualities elicited from the Future Internet Social and Technological Alignment Research (FI-STAR) project by applying the Unified eValuation using ONtology (UVON) method are reported in the Results section, an exploratory result is demonstrated in the Correlation Patterns section, and an estimated model is presented in the Partial Least Squares Structural Equation Modeling Path Models section. The European Union (EU)–wide empirical data collected through the FI-STAR project, detailed in the Methods section and Multimedia Appendix 1, is used to calculate and validate the model in the Partial Least Squares Structural Equation Modeling Path Models section. The results of exploration and model calculations are discussed in the Correlations section and the Partial Least Squares Structural Equation Modeling Path Models section. Subsequently, based on the model, the relative importance of qualities in creating and predicting user satisfaction is discussed in the Most Influential Qualities section. In the section Satisfaction Index, weightings are suggested for the calculation of a satisfaction index for health information systems. Finally, we examine the limits and extensions to our approach and suggested model in the Extensions and Limitations section.

## Methods

### Data Collection

The empirical data for this study have been collected from the FI-STAR project, an EU electronic health (eHealth) project with 7 subprojects across the EU [34]. A convenience sampling approach was used for the recruitment of the participants. Each eHealth app was deployed in a hospital or health facility site and the users on the site—patients or health professionals—were asked about their assessment of the impact of that specific solution on treatment. Participation in the trials, and therefore the survey, was voluntary with no mentioned preconditions. There was no constraint on the type of eHealth project being developed provided that they follow the FI-STAR requirements, especially using the FIWARE infrastructure. However, most of the subprojects could be categorized as telehealth apps. A summary of all subprojects can be found in Multimedia Appendix 1.

### Data Analysis

We applied the UVON method [33] to the FI-STAR requirement documents, together with the evaluation aspects from the Model for ASsessment of Telemedicine framework [35]. The quality aspects appearing in the result of the UVON method are supposed to be provided by the eHealth apps developed in the FI-STAR project. For each quality appearing in the UVON’s output, a question was formulated according to that specific quality in the treatment. The questions were categorized according to the resulting domains in the UVON’s output. The answer alternatives to the questions were formed as a 5-point Likert rating scale with unweighted scores. There were 20 qualities alongside the 3 user satisfaction questions from ECSI [18] that were converted to 2 questionnaires. One questionnaire was customized for the patients and the other one for the health professionals. The content of the questionnaires can be found in Multimedia Appendices 2 and 3. Responses to the questionnaires by 87 patients and 31 health professionals, physicians or nurses, were used for the models suggested in this study.

In 2 steps, we arrived at a model based on the empirical data from the answers to the questionnaires. The calculations were done using the R language, version 3.4.0 [36]. The bootstrapped significance calculation was performed in SmartPLS software version 3.2.7 (SmartPLS GmbH) [37].

**Figure 1:**
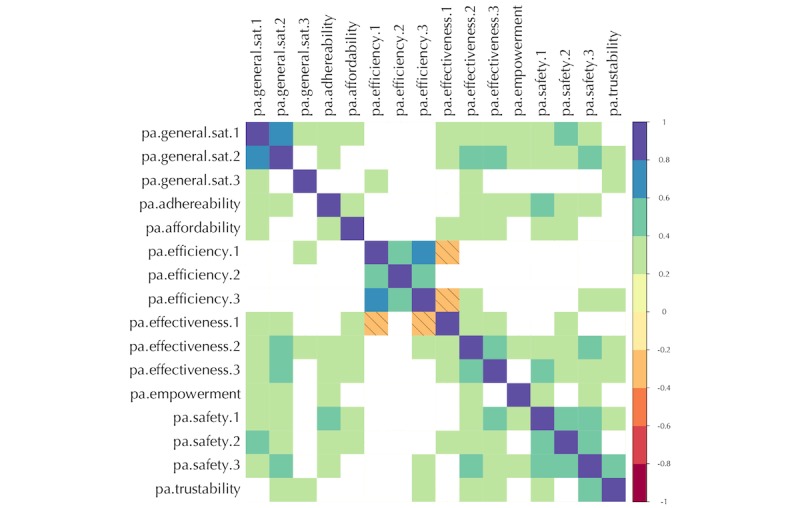
Correlation matrix for the patient questionnaire results across all cases. For the details of each variable, refer to the corresponding question in Multimedia Appendix 2. Insignificant (*P*>.05) results are left blank. Negative results are marked with leftward slanting lines. Note that the qualities grouped by the Unified eValuation using ONtology (UVON) method usually show higher correlations together.

**Figure 2:**
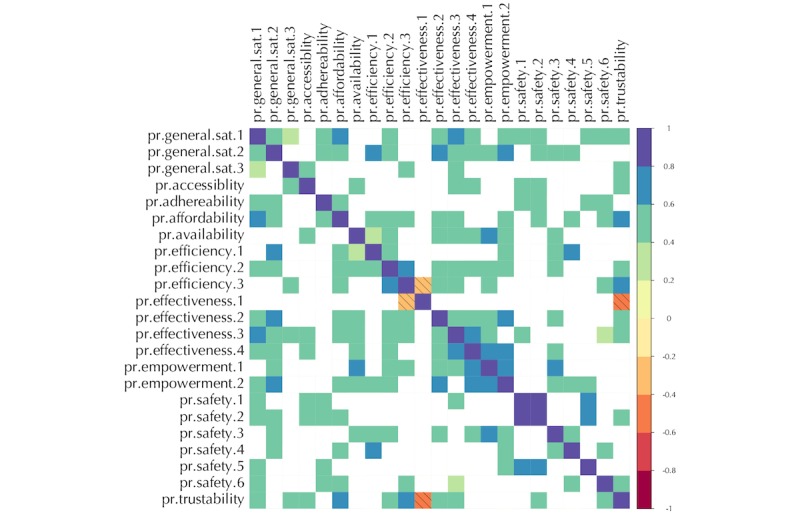
Correlation matrix for the professional questionnaire results across all cases. For the details of each variable, refer to the corresponding question in Multimedia Appendix 3. Insignificant (*P*>.05) results are left blank. Note that the qualities grouped by the Unified eValuation using ONtology (UVON) method usually show higher correlations together.

In the first step, a matrix of Kendall correlation coefficients tau (τ) for each of the patient and professional questionnaires was formed. The results are presented in Figures 1 and 2. Moreover, we used Cronbach alpha (α) test to measure the consistency of the results in the UVON-suggested families of qualities as hints for finding constructs in the later steps.

In the second step, we created a PLS-SEM path model. For each set of the qualities that have already been grouped by the UVON method and show a high degree of correlation, a latent construct variable was considered. These latent variables are not directly measurable but manifest themselves through quality and satisfaction variables. If a quality corresponds only with one question in the questionnaire, one latent proxy variable was considered. Consequently, it would be possible to add more measure variables to the same latent variable in future studies. The PLS-SEM analysis was performed using the *matrixpls* library in R, version 1.0 [38]. The sample size adequacy calculations were performed using G*Power version 3.1 (Heinrich Heine University Düsseldorf), a program for statistical power analysis for a variety of statistical tests [39].

The result of PLS-SEM should be interpreted in the context of the questionnaire. Accordingly, as discussed in the Partial Least Squares Structural Equation Modeling Path Models section, negative coefficients were considered noninformative and were excluded from the final results. The validity of the result was demonstrated through a toolbox of significance, discrimination analysis, internal consistency reliability, and convergence validity. The calculation of significance indicators was performed applying the bootstrapping approach using SmartPLS software [37]. Whenever applicable, the noninformative nature of negative coefficients was considered during the validity and fitness calculations [40].

## Results

### Unified Evaluation Using Ontology Method Outcome

The result of applying the UVON method on the FI-STAR project was a tree-style ontology of qualities [33], of which the top-level qualities are listed in Table 1. The questionnaires articulate those qualities and their more specific subqualities. Table 1 is just an overview of the qualities; details of the questions that were created for each quality can be found in Multimedia Appendices 2 and 3.

Descriptive statistics of the variables in the patient and professional questionnaires, including mean, SD, and median, are shown in Tables 2 and 3. Details of the corresponding question for each quality in Tables 2 and 3 can be found in Multimedia Appendices 2 and 3.

### Correlation Pattern

As can be seen in Figures 1 and 2, a spectrum of weak to strong correlation coefficients appeared in the *Corrgram* diagrams [41] for the patient and professional questionnaire. The blank cells are the results that were not statistically important (*P*>.05). The results of the Cronbach alpha (α) test can be found in Table 4.

**Table 1:** The quality attributes resulting from applying the Unified eValuation using ONtology (UVON) method to Future Internet Social and Technological Alignment Research (FI-STAR) requirement documents.

Quality name	Description	
Accessibility	If the app is accessible to different users	
Adherence	If the patients adhere more to treatment because of the app	
Affordability	If the treatment became more affordable for the patient or health care system because of the app	
Authenticity	If the information provided by the app is authentic and correct (combined with safety)	
Availability	If the service provided by the app is available on demand	
Efficiency	If the treatment is more efficient because the app was used	
Effectiveness	If the treatment process is more effective because the app was used (except for clinical effectiveness)	
Empowerment	If the app empowers the patient or health professional to know more about their conditions or perform their tasks better	
Safety	If the app itself is safe or makes the treatment process safer	
Trustability	If the app improves the trust of the patients in treatment	

**Table 2:** Descriptive statistics of the variables in the patient questionnaire.

Quality^a^	Mean (SD)	Median
pa.adhereability	4.35 (0.73)	4
pa.affordability	4.1 (1.05)	4
pa.effectiveness.1	3.76 (0.94)	4
pa.effectiveness.2	4.28 (0.94)	5
pa.effectiveness.3	4.57 (0.77)	5
pa.efficiency.1	3.82 (0.88)	4
pa.efficiency.2	3.47 (0.9)	3
pa.efficiency.3	3.78 (0.97)	4
pa.empowerment	4.33 (0.9)	5
pa.general.sat.1	4.51 (0.79)	5
pa.general.sat.2	4.46 (0.73)	5
pa.general.sat.3	4.01 (1)	4
pa.safety.1	4.76 (0.46)	5
pa.safety.2	4.58 (0.64)	5
pa.safety.3	4.5 (0.86)	5
pa.trustability	4.62 (0.72)	5

^a^Details of the corresponding question for the items in the Quality column can be found in Multimedia Appendix 2.

**Table 3:** Descriptive statistics of the variables in the professional questionnaire.

Qualitya	Mean (SD)	Median
pr.accessiblity	3.96 (0.88)	4
pr.adhereability	3.91 (0.9)	4
pr.affordability	4.22 (0.8)	4
pr.availability	3.61 (0.99)	4
pr.effectiveness.1	3.39 (0.78)	4
pr.effectiveness.2	4.04 (0.77)	4
pr.effectiveness.3	4.26 (0.81)	4
pr.effectiveness.4	4.26 (0.81)	4
pr.efficiency.1	3.04 (1.02)	3
pr.efficiency.2	3.65 (0.93)	4
pr.efficiency.3	3.91 (1.12)	4
pr.empowerment.1	4.39 (0.58)	4
pr.empowerment.2	4.3 (0.47)	4
pr.general.sat.1	3.87 (1.1)	4
pr.general.sat.2	3.87 (0.97)	4
pr.general.sat.3	3.52 (0.9)	4
pr.safety.1	4.61 (0.58)	5
pr.safety.2	4.57 (0.59)	5
pr.safety.3	4 (0.67)	4
pr.safety.4	3.78 (0.8)	4
pr.safety.5	4.26 (0.69)	4
pr.safety.6	3.96 (0.82)	4
pr.trustability	4.22 (0.74)	4

^a^Details of the corresponding question for the items in the Quality column can be found in Multimedia Appendix 3.

**Table 4:** Cronbach alpha (α) test results for the quality groups.

Quality group		Cronbach alpha (α)^a^
**Patients**		
	pa.general.sat.X	.63
	pa.efficiency.X	.8
	pa.effectiveness.X	.63
	pa.safety.X	.67
**Professionals**		
	pr.general.sat.X	.7
	pr.efficiency.X	.77
	pr.effectiveness.X	.75
	pr.empowerment.X	.82
	pr.safety.X	.79

^a^Although a score over 0.7 is usually considered the desired cut-off criterion, the composite reliability (CR) values in the table numbered 9 can still better determine reliability.

### Partial Least Squares Structural Equation Modeling Path Models

**Figure 3:**
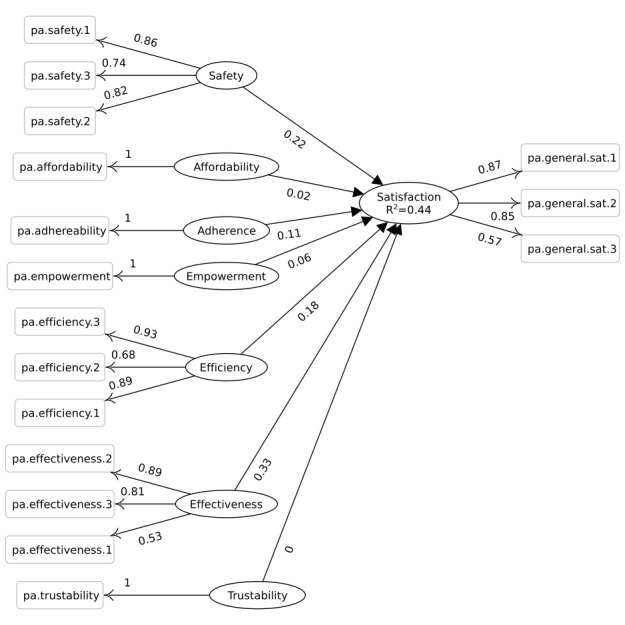
Partial least squares path model for the patient questionnaire. The constructs are shown as ovals, and the number between constructs is the coefficient value. Manifests are shown as rectangles, and the number between a manifest and a construct is the loading value of that manifest.

**Figure 4:**
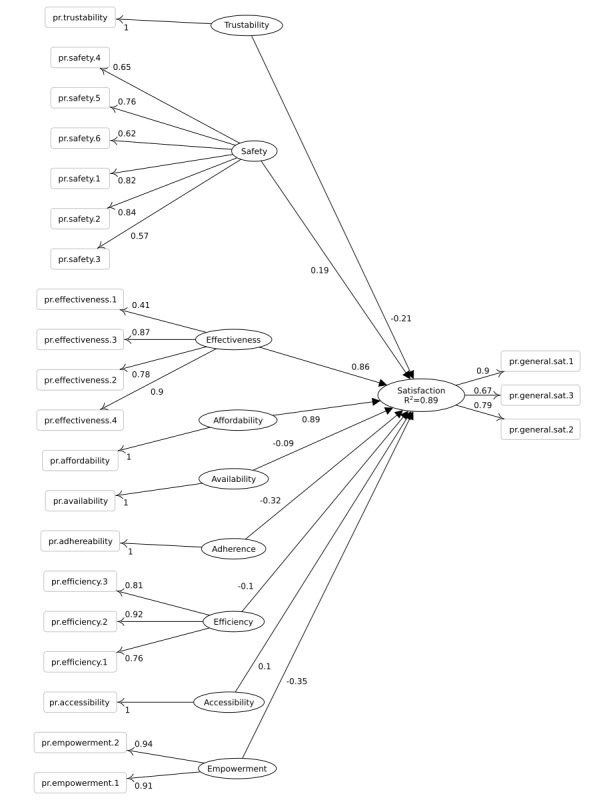
Partial least squares path model for the professional questionnaire. The constructs are shown as ovals, and the number between constructs is the coefficient value. Manifests are shown as rectangles, and the number between a manifest and a construct is the loading value of that manifest.

The 2 PLS-SEM models and their loadings and coefficient values are depicted in Figures 3 and 4. As is common with path models, latent variables are depicted as ovals, whereas manifests are shown as boxes. We considered all the measure variables as reflective, that is, they do not construct their respected latent variables, but they measure or manifest them. The number label on the edge between a manifest and a latent variable is the loading, and the number label on the edge between two latent variables is the coefficient.

The most contributing and predictive qualities regarding satisfaction are reported in Table 5 by specifying the coefficients of relationships between qualities and the satisfaction construct. For the patients, the coefficients of effectiveness, safety, and efficiency qualities were higher than the average (.13) of all coefficients. Similar to the professional, coefficients of the affordability and effectiveness qualities were higher than the average (.51) of all coefficients.

The relationship of each measure variable in the path model with its construct is associated with weights. The standardized weights for the satisfaction construct measure variables are required for calculating the *user satisfaction index* and can be found in Table 6.

**Table 5:** The coefficients of the qualities to satisfaction relationships in the partial least squares structural equation modeling path model show which qualities contribute more to satisfaction.

Quality construct^a^	Patient coefficient	Professional coefficient
Effectiveness	.33	.86
Safety	.22	.19
Affordability	.02	.89
Efficiency	.18	—^a^
Adherence	.11	—
Empowerment	.06	—
Trustability	0	—
Accessibility	—	.1

^a^— represents negative values as being noninformative (see section Partial Least Squares Structural Equation Modeling Models). For the conclusion, one should consider the significance, as shown in table numbered 7.

**Table 6:** Standard weights for calculating the satisfaction index, based on the manifest variable loadings for the Satisfaction constructs in the patient and professional path models.

Manifest variable		Standardized weight
**Patients**		
	pa.general.sat.1	0.36
	pa.general.sat.2	0.38
	pa.general.sat.3	0.25
**Professionals**		
	pr.general.sat.1	0.4
	pr.general.sat.2	0.32
	pr.general.sat.3	0.28

**Table 7:** Significance of the quality to satisfaction relationships by calculating the *P* values of the relationships between the qualities and the Satisfaction construct. If a relation does not exist in model, the corresponding cell in the table is left blank.

Antecedent to Satisfaction	Patient *P* value	Professional *P* value
Adherance	.16	.13
Affordability	.38	.04^a^
Effectiveness	.01^a^	.01^a^
Efficiency	.03^a^	.37
Empowerment	.25	.20
Safety	.04^a^	.38
Trustability	.48	.30
Accessibility		.41
Availability		.42

^a^*P* values<.05.

The *P* value results of calculating the significance of quality to success relationships using the bootstrapping approach are shown in Table 7. The *P* values below .05 are marked with footnotes. Regarding discriminant analysis, the results of the Heterotrait-Monotrait (HTMT) ratio are demonstrated in Table 8. HTMT being below 1.0, preferably 0.9, satisfies the discriminatory criterion [42]. In addition, Multimedia Appendix 4 depicts cross-loadings in the path models. The effect sizes of the samples were enough to show significant results for the highest loading constructs, as shown in Table 5. Details of the effect sizes and their associated power, by recalculating the PLS-SEM and focusing on significant relations, are shown in Multimedia Appendix 5. The list of acronyms is provided in Multimedia Appendix 6.

**Table 8:** The discriminant validity analysis shows if the manifests of a construct in the patient or professional Partial Least Squares Structural Equation Modeling models have the strongest relationship with that construct compared with another construct. If a relation does not exist in model, the corresponding cell in the table is left blank.

Construct pairs (A vs B)^a^	Patient HTMT^b^	Professional HTMT
Efficiency→Satisfaction	0.21	0.84
Effectiveness→Satisfaction	0.79	1.07
Safety→Satisfaction	0.69	0.91
Effectiveness→Efficiency	0.04	0.68
Safety→Efficiency	0.09	0.62
Safety→Effectiveness	0.75	0.68
Empowerment→Satisfaction		0.69
Empowerment→Efficiency		0.76
Empowerment→Effectiveness		0.97
Safety→Empowerment		0.63

^a^Heterotrait-Monotrait ratio results below 1.0, preferably 0.9, satisfy the discriminatory criterion.

^b^HTMT: Heterotrait-Monotrait.

**Table 9:** The result of internal consistency reliability of the manifest variables by calculating composite reliability and their convergence by measuring average variance extracted, grouped by constructs.

Construct^a^	CR^b^ patient	AVE^c^ patient	CR professional	AVE professional
Satisfaction	0.82	0.6	0.83	0.63
Adherence	1	1	1	1
Affordability	1	1	1	1
Efficiency	0.88	0.71	0.87	0.7
Effectiveness	0.8	0.57	0.84	0.58
Empowerment	1	1	0.92	0.85
Safety	0.85	0.66	0.86	0.51
Trustability	1	1	1	1
Accessibility			1	1
Availability			1	1

^a^A composite reliability value above 0.7 and an average variance extracted value above 0.5 are preferred.

^b^CR: composite reliability.

^c^AVE: average variance extracted.

For internal consistency reliability, composite reliability (CR) values, and for convergence validity, average variance extracted (AVE) values were calculated for each construct, as depicted in Table 9. The minimum CR should preferably be above 0.7 [43]. The minimum AVE should preferably be above 0.5 [44].

## Discussion

### Overview

This study advances and prioritizes the qualities in health information systems that determine and predict user satisfaction, both for patients and health professionals. As a secondary outcome, it also suggests weightings for calculating the *satisfaction index*. The outcomes of the study exhibit the effectiveness of the UVON method in proposing quality constructs that can be applied to a path analysis. Conclusions from the results are achieved in 2 steps. First, the correlations give better insight about the groupings of the qualities as manifest variables of the latent constructs. Second, the path model justifies and quantifies the relationship between those grouped qualities, their latent construct variables, and the satisfaction construct.

### Correlations

In the exploratory step, as shown in Figures 1 and 2, strong correlations appear between the qualities that have been already grouped into a family by the UVON method. These high correlations result from the semantic unification of qualities across branches of an ontological tree by the UVON method [33]. For example, a set of above-medium correlations exist within the efficiency, effectiveness, and safety family of qualities in the patient questionnaire results, as well as within the empowerment, effectiveness, safety, and efficiency families in the professional questionnaire results. The same is true for the satisfaction questions in both groups of questionnaires.

The above correlations suggest that the members of a quality or satisfaction group can be combined. Alternatively, in other words, they manifest a common latent variable. It is worth mentioning that the Kendall correlation tau (τ) is less generous than Spearman rank correlation rho (ρ) in confirming the correlations [45]. Hence, there would be more confidence in positively interpreting the correlation results and suggesting a common latent origin. The Cronbach alpha results in Table 4 confirm the same explanation in the quality groups.

Besides the possible existence of latent variables, there are 2 other corollaries to the correlations. First, the high degree of correlation between a family of qualities results in the multicollinearity problem. Multicollinearity makes ordinary regression techniques inefficient and the interpretation of the regression coefficients challenging [46]. Overall, 2 solutions can be taken here: choose one of the variables that show high correlation with each other by using variable selection methods, such as Least Absolute Shrinkage and Selection Operator, or apply a method that is tolerant to the problem. The PLS-SEM approach used in this study is tolerant to multicollinearity; meanwhile, it can investigate the causality relations between some correlated quality groups.

Second, the correlation between qualities and satisfaction aspects suggests a causality relationship between quality and user satisfaction. Similarly, there are models, such as the CSI family of models [11-13] as well as the D&M IS model [20], that demonstrate a causality relationship between qualities and satisfaction in parts of their structure. We can draw on corroborations from the extensive amount of literature about those models, both to enrich our model and verify the results.

A summary of the above discussion is that we can group the qualities within a family as manifests of a latent variable, consider a causal relationship from those quality latent variables to a satisfaction latent variable, and present these groupings and relationships through a PLS-SEM model.

### Partial Least Squares Structural Equation Modeling Path Models

The PLS-SEM path model has traditionally been used to represent causalities for the CSI series [11,12,47], the models related to D&M IS [48,49], and similar intentions [50]. This prevalence of usage gives the opportunity to reuse some of those models’ parts, compare their structures, and collate their results. Other advantages of the PLS-SEM approach are the need for small sample size and the ability of handling non-normal data [50].

The 2 PLS-SEM path models in this study comply with the general pattern in CSI, D&M IS, and TAM series models in which a central construct—be it called system success, user satisfaction, customer satisfaction, or user acceptance—is influenced by system qualities. Besides the use of different constructs, each model captures a distinct level of detail for the same or similar concepts. The CSI and TAM models are more concerned about the general perception of quality, whereas D&M IS examines further details about the qualities by considering 3 separate constructs: *system quality*, *information quality*, and *service quality*. The model presented in this study is inclined to be more domain-specific by focusing on the health information system domain. The model is also more concerned with the qualities improved in the whole treatment setting by using the health information system rather than the qualities of the system. Finally, in comparison with the previously mentioned mainstream models, the model in this study is more specific about the type of qualities and how they compare in determining and predicting user satisfaction.

Before discussing the qualities with the most influence on satisfaction, model validity and the right way of interpreting the results need to be investigated.

Regarding internal consistency reliability, CR indicators need to be higher than 0.7 [43], which is well satisfied (see Table 9). The CR shows if the manifest variables of each construct measure the same thing. The convergent indicator AVE needs to be more than 0.5 to indicate that more than half of the variance in the measures is because of the variance in the construct [44]. All the constructs in our PLS-SEM model satisfy this criterion (refer to Table 9).

From the discriminatory validation perspective, both patient and professional models show indications of correctly assigning the measure to construct variables. The HTMT ratio demonstrates if the assignment of measuring variables to a specific construct is better, that is, more relevant, than other alternatives. All the HTMT results in both models, except one as shown in Table 8, satisfy the specified criterion of being less than 1. Furthermore, most of the HTMT values are less than 0.9, which confirms discriminant validity [42]. The only pair of constructs that have a ratio value above 1 is the *effectiveness* and *satisfaction* pair for professionals. However, this unfulfilled criterion might be justified considering that, in the domain, one should segregate satisfaction and effectiveness, while effectiveness highly contributes to satisfaction. The relatively high HTMT ratio for the pair of *empowerment* and *effectiveness* can indicate that the users’ empowerment to reach effectiveness is not very distinctive from the improved effectiveness.

The interpretation of the negative coefficients that appeared in the models must be discussed. The wording in the FI-STAR questionnaires captures only the user perspective on positive relationships but not the negative ones. The information gathered from the questions is unidirectional. Therefore, one cannot interpret the negative coefficients as an indication that some qualities are inversely related to satisfaction.

For example, if it is asked whether the app has increased the effectiveness of a treatment by decreasing the number of mistakes, a responder might answer “disagree.” This answer can mean whether the user does not believe that the app has decreased the number of mistakes in the treatment or the user might think that the app has decreased the number of mistakes but does not contribute to the system’s effectiveness. On the other hand, it is still a possible interpretation that the app has caused more mistakes; however, we cannot separate this interpretation from the other previously mentioned valid interpretations. Therefore, we can only confirm the positive part of relationships, where quality contributes to user satisfaction.

### Most Influential Qualities

As shown in Table 7, there are constructs whose relationships to the satisfaction construct are statistically significant. Those constructs also have a considerable impact on satisfaction, as depicted in Table 5. Regarding satisfaction as the major contributor to success [51] and the indicator of voluntary acceptance [14], we extend our discussion to cover similar studies that report on these 2 indicators.

Within the list of qualities in Table 7 with a significant relationship to the satisfaction construct, the degree of *effectiveness* is considerably predictive in creating satisfaction. Both patients and professionals care considerably whether an app has increased the effectiveness of treatment and care. This result highlights the nonintuitive contrast between the effectiveness of other qualities, such as the efficiency for patients, in affecting their satisfaction. Nevertheless, there can be alternative interpretations. For example, if the apps in the FI-STAR project could significantly improve efficiency for patients, efficiency might have shown an impact as the effectiveness on satisfaction.

This result confirms the studies that consider effectiveness the major contributor or even equivalent to user satisfaction, generally, in information systems [14,51,52]. More specifically, this study parallels the studies that reported effectiveness (sometimes expressed as usefulness) as the most, or one of the most, influential qualities for the satisfaction of patients [53] or health professional users [54-56] in a variety of health information systems. Nevertheless, there exist studies that reached a different conclusion in prioritizing the most influential qualities [57].

It should be noted that improvements in the *effectiveness* of the treatment are not articulated identically in all those studies. For comparison, one needs to consider this discrepancy [8]. Some studies have used *performance* [54], a term presumably borrowed from the D&M IS and TAM families. In addition, some studies reported similar manifest variables to effectiveness in our study—see Multimedia Appendices 2 and 3 —such as making fewer mistakes [58].

According to our results, *affordability* has a high degree of impact on satisfaction for professionals, similar to effectiveness. The affordability to satisfaction relationship for the patients was not statistically significant in this study, whereas its magnitude was also negligible compared with other relations. An explanation might be that, in the FI-STAR setting, patients did not have to be concerned about the costs and affordability of solutions, whereas professionals might have a more holistic perspective. In a different setting, where patients are more concerned about treatment costs, their satisfaction might be more influenced by the improvements in affordability, showing higher significance and magnitude in the coefficient that relates affordability to satisfaction constructs.

Although studies report increased affordability and cost reduction in treatment can improve the satisfaction and acceptance of health information systems [2,59], only some of these studies quantify or compare the degree that affordability and cost reduction affect satisfaction. In addition, many studies such as ours could not report definitively how patients perceived cost-reduction quality considering patients usually do not pay for treatment in the context of a study [5,60]. Some studies that rely on TAM models have considered affordability, alongside other factors, as a manifest to *perceived usefulness* [8,61,62]. These studies show a relatively high or above-average impact on professional users’ satisfaction, acceptance, or intention to use [61,62]. Our results in Table 5 comply with these studies. It is worth noting that some studies declared the same idea in a negative form, where being costly is considered a barrier to acceptance [2,60,63] or success [7].

Patients showed some degree of improved satisfaction when there was an improvement in *efficiency* or *safety*. It is important to note that health information systems, as a side effect, can degrade the status of efficiency or safety in a health care setting [64]. Hence, their contribution to overall satisfaction can even be negative. However, as highlighted before, our questionnaires were not designed to differentiate between the states of negative impact and no impact.

Considering most of the apps in the FI-STAR project could be categorized as telemedicine apps (see Multimedia Appendix 1), it could be predicted that efficiency, achieved by eliminating the hassle of distant travels, contributes to user satisfaction to some extent. Despite our initial expectations, the degree of impact on satisfaction, although existing, was less than the previously mentioned factors. Our expectation was based on similar studies that investigated the impact of efficiency improvements on satisfaction and acceptance: the degree of impact was recorded relatively more than our study [1]. It is also important to note that we considered a separate category for affordability and cost saving quality, whereas some studies considered cost saving a form of improving efficiency [65] or a manifest to *perceived usefulness* [62]; therefore, other results should be compared with more attention to this detail. From the other side, some studies considered other forms of efficiency, rather than affordability, as manifests to *perceived usefulness* [55,66,67]. Regarding the coefficients and loadings in models of the aforementioned studies, in comparison with our results, they have recorded a higher impact from efficiency on the satisfaction or acceptance of users.

Looking at the safety questions for the patients in Multimedia Appendix 2, it seems that being informed about the situation and capable of keeping the situation in check is the source of the safety to satisfaction causality. Similar to other qualities, safety has been categorized in various constructs in the studies, whether as a manifest to *provider performance* [68], *perceived usefulness* [62], *outcome* [62], or *information satisfaction* [62].

To the best of our knowledge, there are few studies that investigate the impact of safety brought by health information systems on the satisfaction or acceptance of the users of those systems. However, one should pay attention to this caveat that the *safety* concept might have divergent embodiments in various studies. Two of the manifest variables in our models, *pa.safety.2* and *pr.safety.3*, refer to providing correct information, which is also mentioned in distinct studies, mostly as a manifest variable for the *information quality* construct [26,58,69]. In these studies, providing the correct information influences satisfaction or acceptance relatively high or above average. Moreover, there are some studies on the systems in which their primary function is to improve safety aspects. As can be anticipated, they report a high impact from safety on the intention to use [70,71].

### Satisfaction Index

Each of the satisfaction constructs in the PLS-SEM models is operationalized by 3 measure variables, embodied as 3 questions. The relationship of each of these measure variables with the latent satisfaction construct is characterized by loading and weighting values. The weighting values make it possible to calculate a weighted *satisfaction index*, both for patients and professionals. Using this index can be a makeshift way of assessing qualities inside health information systems.

The standardized weightings for the scores of the 3 measure questions of satisfaction are determined by the overall balance in the model as it is implemented by the PLS-SEM algorithm. The weightings, along with the scores of those 3 questions, make it possible to calculate the user satisfaction that is engendered by the improvement of qualities. Without the weightings, the satisfaction score represents the evaluation of a kind of unidentified trait that is relevant but not necessarily the same as a quality engendered satisfaction. A larger and more diverse sample population of respondents and apps might be needed to stabilize the weightings for a larger scope.

Collecting satisfaction scores is a common practice in health-related studies. However, to the best of our knowledge, the studies on health information systems that suggest a kind of satisfaction index with adjusted weights regarding other qualities are limited. Conversely, the studies that have calculated the parameters of TAM or D&M IS models in their contexts or have used a path model that includes user satisfaction alongside other impacted qualities, such as studies by Jo et al, Schaper et al, and Pai et al [53,56,69], implicitly suggest a kind of indexing for adoption and satisfaction aspects. Nevertheless, there are barriers to utilizing the measures suggested for the satisfaction construct. First, the extent and diversity of subjects in these studies are important factors to reuse their suggested measures and their associated weights. Second, using arbitrary measure questions or the number of items for measurement can create a burden beyond a study’s resources. A sample of this case is the End User Computing Satisfaction measures that range from 12 to 39 items [52,72]. Although some studies have the required resources to apply them [25], using those instruments is not feasible in many other cases. Our focus on the 3 standard satisfaction measures from CSI makes it easier to implement the study and simultaneously facilitates the running of interdisciplinary comparisons and knowledge about the satisfaction of users (customers) of health information systems and other services and products.

The qualities investigated in this study can explain different amounts of satisfaction variations with *R^2^*=.43 for patient satisfaction and *R^2^*=.88 for professional satisfaction (as depicted in Figures 3 and 4). The satisfaction index can facilitate an informed guess about the qualities when the user perspectives on those qualities are missing or hard to elicit. This approach is a makeshift way to evaluate the qualities improved by a system. A practical application might be to use the satisfaction index when comparing a pair of similar systems in similar contexts. Another application is to compare the past and present state of the same system that has undergone quality improvement, but no other system or environmental aspect has been changed. Generally, if there are similarities in context and functionalities, and there has been no drastic change or difference in qualities improved by systems, the satisfaction index can serve as a good indicator for an informed guess about those qualities.

### Extensions and Limitations

The list of most influential qualities should be read with the precaution of how the similar or even the same qualities have been articulated differently in studies [73]. Studies that recruit highly cited frameworks also tend to recruit similar wordings for the qualities. However, other studies practice their freedom to use the wording that best matches their case, resulting in divergent wordings for similar concepts. In our case, notably, the improvement of a treatment’s *effectiveness* is largely similar to *performance* or *performance expectancy* in the studies that are based on the TAM family, and consider the *performance expectancy* definition as “the degree to which an individual believes that using the system will help him or her to attain gains in job performance” [74]. It is similar to what we asked about effectiveness (refer to Multimedia Appendices 2 and 3). However, one can still find research based on TAM studies that used *performance expectancy* as a form of efficiency [29]. In addition, *efficiency* in our study is more or less similar to *effort expectancy* or *perceived usefulness* in the TAM series. Some other studies have used *productivity* or even *performance* instead of what we called efficiency [68], and others considered efficiency as a manifest to *perceived usefulness* [8,55,66,67].

Another caveat when comparing the results is that the qualities specified in our study are the qualities of the treatment or care that using FI-STAR systems might have improved, which are different from the intrinsic qualities of those systems. Accordingly, for example, a higher speed system might increase the affordability of treatment and care in some way. What we have focused on was the affordability of care but not the speed of the system. Therefore, reports on the intrinsic system qualities that have increased user satisfaction or acceptance cannot be compared directly with our study, unless those qualities get translated to their final impact on treatment and care.

All the eHealth apps, being developed in the FI-STAR project, were supposed to support the required general technical specifications, which includes using the FIWARE infrastructure and being based on *software to data* paradigm [75]. These requirements were not constraints on the diversity of apps as indicated in Multimedia Appendix 1. Nevertheless, the diversity of the apps should be considered when generalizing the results of this study. For example, if the main outcome of an app is improving the safety of treatment, users might consider both safety and effectiveness almost the same; however, in other apps, they can consider them as distinctive qualities. As another example, the effect of some apps on affordability of treatment can be varied in a different context, when the patients pay for the treatment. This study relies on the output of the UVON method, which extracts common qualities between a set of apps; however, as it is shown in abovementioned examples, user perspectives on those common qualities could be diverse. Therefore, generalization of the results of this study should be done bearing this caveat in mind.

The predictive power of qualities in projecting user satisfaction can support design decision making for health information systems. When trade-offs are necessary, designers can prioritize features if they can compare their user satisfaction yield. Knowing the quality profile of each feature, one can combine that with the table of quality to satisfaction magnitudes, such as in Table 5, to arrive at more informed feature selection decisions [76].

Another extension to the model of this study is to consider the qualities or system usage ramifications that impact satisfaction negatively. This needs articulation of questions to capture negative attitudes—not noninformative ones—about the impacts of systems. That kind of wording permits elicitation of constructs that are negatively related to other constructs or new manifest variables for the current constructs that reflect the construct negatively.

A possible future extension to our PLS-SEM model is to consider relationships between quality latent variables. In the model presented in this study, no relationship has been proposed between the quality constructs, but one might try, for example, to investigate if a system that improves adherence also changes user attitudes about its contribution to effectiveness. However, considering that only recursive relationships can be used in the PLS-SEM models, we cannot investigate the circular impact between qualities and satisfaction with this technique [77,78].

### Conclusions

The satisfaction of health information system users is highly influenced by certain qualities that are improved by those systems. Both patient and professional users consider improvements to the effectiveness of health care a highly important quality that makes them satisfied with the system. For patient users, safety and efficiency qualities come after effectiveness in creating satisfaction. For health professionals, better health care affordability brought by health information systems is important, much like effectiveness, in creating their satisfaction.

The PLS-SEM model presented in this study can demonstrate the above ranking of qualities in the creation of user satisfaction. Furthermore, the model suggests weightings to calculate the *satisfaction index* for health information systems. The satisfaction index can be used to compare and monitor health information systems from user satisfaction and quality improvement perspectives.

## Appendix

Multimedia Appendix 1 Summary of the FI-STAR trial cases.

Multimedia Appendix 2 Questionnaire for the patients.

Multimedia Appendix 3 Questionnaire for the health professionals.

Multimedia Appendix 4 Crossloadings in the path models.

Multimedia Appendix 5 Effect size and power analysis.

Multimedia Appendix 6 Acronyms.

## Abbreviations

AVE: average variance extracted
CR: composite reliability
CSI: Customer Satisfaction Index
D&M IS: Delone and McLean Information Systems Success
ECSI: European Customer Satisfaction Index
eHealth: electronic health
EU: European Union
FI-STAR: Future Internet Social and Technological Alignment Research
HTMT: Heterotrait-Monotrait
PLS-SEM: partial least squares structural equation modeling
TAM: technology acceptance model
UVON: Unified eValuation using ONtology
