# Supplementary material for: Most Influential Qualities in Creating Satisfaction Among the Users of Health Information Systems: Study in Seven European Union Countries
Source: JMIR Med Inform. 2018 Nov 30;6(4):e11252. doi: 10.2196/11252 (PMC6294876; doi:10.2196/11252)
Supplement: Multimedia Appendix 1 [file medinform_v6i4e11252_app1.pdf]

Appendix A: Summary of the FI-STAR trial cases

In Tromsø, Norway, two mobile apps — integrated at the backend — were developed to record biometric parameters and provide remote counselling and community services. In Krakow, Poland, a tele-care cloud solution monitors both preoperative rehabilitation in thoracic-surgical treatments and the physical and psychological state of patients during chemotherapy. In Munich, Germany, a surgical operating theatre monitoring system tracks and reports the consumables used during an operation. No patient questionnaire was delivered for this case. In Bilbao, in the Basque country of Spain, a telemedicine cloud solution provides treatment management for patients with bipolar disorder. In Bucharest, Hungary, a telemedicine cloud solution provides at-home cardiac rehabilitation services by monitoring and measuring biometrics. In Bologna, Italy, a telemedicine cloud solution assists in the treatment of chronic obstructive pulmonary disease (COPD) by collecting and reporting patients' vital parameters, and providing those parameters through tele-monitoring to medical personnel. One of the FI-STAR trial cases was being held in Leeds in the United Kingdom (UK), to provide a cloud solution for ensuring drug authenticity and preventing dispensing errors. The requirement documents from this case contributed to the creation of the questionnaires, but the data was not available in the evaluation phase. Accordingly, we could not use it as an input for our study. There was also a late-joining trial in Munich in which its requirements were not included during the creation of the questionnaires, but the users eventually participated in answering them. The details of each trial case can be found in the EU CORDIS database [34] or FI-STAR project websites [78].
