# Supplementary material for: Most Influential Qualities in Creating Satisfaction Among the Users of Health Information Systems: Study in Seven European Union Countries
Source: JMIR Med Inform. 2018 Nov 30;6(4):e11252. doi: 10.2196/11252 (PMC6294876; doi:10.2196/11252)
Supplement: Multimedia Appendix 2 [file medinform_v6i4e11252_app2.docx]

Appendix B: Questionnaire for the patients

1. Satisfaction

    a. pa.general.sat.1: How satisfied are you with the application's health care output in its entirety?

    b. pa.general.sat.2: How well the application fulfills your expectations in health care delivery?

    c. pa.general.sat.3: Imagine a perfect application in all aspects of health care delivery. How far away from that is the application you are using today?

2. Adherence

    a. pa.adherability: The application increased my motivation for the treatments

3. Affordability

    a. pa.affordability: The health care service delivered through the application is more affordable or decrease expenditures, comparing with other alternatives.

4. Efficiency: If you have experienced the same treatment before, would you say that the application has increased efficiency by reducing:

    a. pa.efficiency.1: Complexity or number of tasks

    b. pa.efficiency.2: Number of reworks

    c. pa.efficiency.3: Time consumed

5. Effectiveness: The application has increased effectiveness, especially by improving at least one of these items:

a. pa.effectiveness.1: Less mistakes

b. pa.effectiveness.2: Readiness or promptness for different situations

c. pa.effectiveness.3: More personalized treatment

6. Empowerment

   a. pa.empowerment.3: The application empowers me by increasing my knowledge about the situation or general knowledge about the disease.

7. Safety

   a. pa.safety.1: I felt safe when using the application.

   b. pa.safety.2: The application provides correct information without any mislead or confusion.

   c. pa.safety.3: I felt that the application helped me to minimize possible harms during the usage.

8. Trustability:

   a. pa.trustability I felt that I can trust the application for my privacy and my information.
