# Supplementary material for: Most Influential Qualities in Creating Satisfaction Among the Users of Health Information Systems: Study in Seven European Union Countries
Source: JMIR Med Inform. 2018 Nov 30;6(4):e11252. doi: 10.2196/11252 (PMC6294876; doi:10.2196/11252)
Supplement: Multimedia Appendix 3 [file medinform_v6i4e11252_app3.docx]

Appendix C: Questionnaire for the health professionals

1. Satisfaction

   a. pr.general.sat.1: How satisfied are you with the application's health care output in its entirety?

   b. pr.general.sat.2: How well the application fulfills your expectations in health care delivery?

   c. pr.general.sat.3: Imagine a perfect application in all aspects of health care delivery. How far away from that is the application you are using today?

2. Accessibility

   a. pr.accessibility: The application is easily accessible for different groups of users

3. Adherence

   a. pr.adherence: The application helps patients to more adherence by some sort of motivators (i.e. being a member of a community)

4. Affordability

   a. pr.affordability: The health care service delivered through the application is more affordable or decrease expenditures, comparing with other alternatives.

5. Availability

   a. pr.availability: The service which is provided by the application, is available on demand

6. Efficiency: The application has increased efficiency by reducing

   a. pr.efficiency.1: Complexity or number of tasks
   b. pr.efficiency.2: Number of reworks
   c. pr.efficiency.3: Time consumed
7. Effectiveness: The application has increased effectiveness, especially by improving at least one of these items:
   a. pr.effectiveness.1: Less mistakes
   b. pr.effectiveness.2: Readiness or promptness for different situations
   c. pr.effectiveness.3: More knowledge and evidences
   d. pr.effectiveness.4: More personalized treatment
8. Empowerment
   a. pr.empowerment.1: The application empowers the patient by increasing their knowledge about their situation or general knowledge about the disease.
   b. pr.empowerment.2: The application empowers the medical personnel by increasing their knowledge about the patient situation or general knowledge about the disease.
9. Safety
   a. pr.safety.1: It is safe for the patients to use the application without any possibility of disability, morbidity, or mortality harm.
   b. pr.safety.2: It is safe for the medical personnel to use the application without any possibility of disability, morbidity, or mortality harm.

c. pr.safety.3: The application provides correct information without any mislead or confusion.

d. pr.safety.4: The application provides enough information on how to minimize possible harms during the usage

e. pr.safety.5: If the application fails, and if the failure causes any harm, then the harm would be minor as it would be non-severe, rarely happening, or happening for a very short duration.

f. pr.safety.6: The application improves health care safety by detecting emergency situations, unsafe behaviors or glitches in the process.

10. Trustability

a. pr.trustability.1: The application attains trust of patients (for example by ensuring privacy of their information or being non-invasive in its interaction with them)
