# Supplementary material for: Most Influential Qualities in Creating Satisfaction Among the Users of Health Information Systems: Study in Seven European Union Countries
Source: JMIR Med Inform. 2018 Nov 30;6(4):e11252. doi: 10.2196/11252 (PMC6294876; doi:10.2196/11252)
Supplement: Multimedia Appendix 4 [file medinform_v6i4e11252_app4.docx]

Appendix D: Crossloadings in the path models

Table 1. Cross-loadings for the patients path model.

	ADHEREABILIY	AFFORDABILITY	EFFECTIVENESS	EFFICIENCY	EMPOWERMENT	SAFETY	SATISFACTION	TRUSTABILITY
pa.adhereability	1	0.28	0.41	0.22	0.29	0.59	0.44	0.22
pa.affordability	0.28	1	0.4	0.16	0.07	0.24	0.27	-0.1
pa.effectiveness.1	0.12	0.28	0.53	-0.23	-0.11	0.18	0.22	-0.17
pa.effectiveness.2	0.31	0.37	0.89	0.33	0.24	0.48	0.57	0.16
pa.effectiveness.3	0.47	0.25	0.81	0.21	0.17	0.55	0.38	0.11
pa.efficiency.1	0.19	0.08	0.22	0.91	0.17	0.18	0.3	0.18
pa.efficiency.2	0.03	0.19	0.04	0.67	0.13	0.07	0.15	0.19
pa.efficiency.3	0.27	0.17	0.26	0.93	0.13	0.27	0.38	0.24
pa.emp owerme	0.29	0.07	0.19	0.17	1	0.3	0.26	0.07

nt						5		
pa.general.sat.1	0.42	0.33	0.45	0.22	0.25	0.5	0.87	0.07
pa.general.sat.2	0.36	0.2	0.51	0.31	0.3	0.45	0.85	0.12
pa.general.sat.3	0.22	0.07	0.34	0.32	0.01	0.28	0.58	0.28
pa.safety.1	0.62	0.22	0.47	0.19	0.36	0.86	0.4	0.2
pa.safety.2	0.49	0.25	0.5	0.1	0.22	0.82	0.45	0.08
pa.safety.3	0.33	0.12	0.39	0.26	0.27	0.74	0.45	0.51
pa.trustability	0.22	-0.1	0.1	0.24	0.07	0.33	0.18	1

Table 2. Cross-loadings for the professional path model.

	**ACCESSABILITY**	**ADHERABILITY**	**AFFORDABILITY**	**AVAILABILITY**	**EFFECTIVENESS**	**EFFICIENCY**	**EMPOWERMENT**	**SAFETY**	**SATISFACTION**	**TRUSTABILITY**
pr.accessibli	1	-	0.21	0.56	0.44	0.2	0.34	0.3	0.44	0.51

ty		0.01				6		9		
pr.adhereability	-0.01	1	0.66	-0.14	0.29	0.35	0.26	0.58	0.45	0.3
pr.affordability	0.21	0.66	1	0.29	0.34	0.62	0.42	0.64	0.73	0.69
pr.availability	0.56	-0.14	0.29	1	0.63	0.52	0.6	0.23	0.51	0.37
pr.effectiveness.1	-0.11	-0.01	-0.22	0.21	0.41	-0.06	0.29	-0.02	0.22	-0.47
pr.effectiveness.2	0.34	0.34	0.43	0.56	0.78	0.55	0.63	0.46	0.67	0.39
pr.effectiveness.3	0.46	0.28	0.33	0.53	0.87	0.54	0.54	0.43	0.67	0.51
pr.effectiveness.4	0.4	0.16	0.19	0.53	0.9	0.47	0.73	0.45	0.58	0.21
pr.efficiency.1	0.15	0.3	0.55	0.42	0.3	0.76	0.49	0.51	0.45	0.17
pr.efficiency.2	0.37	0.23	0.47	0.54	0.57	0.92	0.62	0.45	0.5	0.38

pr.efficiency.3	0.13	0.35	0.53	0.34	0.55	0.81	0.45	0.26	0.52	0.57
pr.empovterment.1	0.39	0.07	0.2	0.67	0.75	0.6	0.91	0.39	0.45	0.22
pr.empovterment.2	0.25	0.39	0.54	0.46	0.62	0.56	0.94	0.58	0.55	0.19
pr.general.sat.1	0.32	0.45	0.71	0.41	0.64	0.47	0.45	0.61	0.9	0.49
pr.general.sat.2	0.26	0.56	0.63	0.42	0.64	0.63	0.65	0.66	0.79	0.36
pr.general.sat.3	0.49	0	0.34	0.39	0.5	0.29	0.16	0.32	0.67	0.65
pr.safety.1	0.5	0.54	0.39	0.12	0.32	0.24	0.27	0.82	0.44	0.42
pr.safety.2	0.4	0.61	0.5	0.09	0.27	0.25	0.26	0.84	0.51	0.44
pr.safety.3	0.31		0.25	0.48	0.5	0.47	0.68	0.57	0.51	0.28
pr.safe	-0.01	0.29	0.51	0.23	0.32	0.4	0.47	0.	0.45	0.16

ty.4						4		65		
pr.safety.5	0.4	0.48	0.39	-0.04	0.24	0.04	0.36	0.76	0.36	0.33
pr.safety.6	0.12	0.55	0.64	0.03	0.41	0.51	0.23	0.62	0.55	0.54
pr.trustability	0.51	0.3	0.69	0.37	0.36	0.46	0.22	0.52	0.61	1
