# Supplementary material for: Most Influential Qualities in Creating Satisfaction Among the Users of Health Information Systems: Study in Seven European Union Countries
Source: JMIR Med Inform. 2018 Nov 30;6(4):e11252. doi: 10.2196/11252 (PMC6294876; doi:10.2196/11252)
Supplement: Multimedia Appendix 5 [file medinform_v6i4e11252_app5.docx]

Appendix E: Effect size and power analysis

Table 1. Effect size and power of the quality to success relationships.

Antecedent to Satisfaction	Patient f^2	Patient power	Professional f^2	Professional power
AFFORDABILITY			1.51	> 0.99
EFFECTIVENESS	0.14	0.93	1.66	> 0.99
EFFICIENCY	0.07	0.67		
SAFETY	0.10	0.84		
