# Supplementary material for: Most Influential Qualities in Creating Satisfaction Among the Users of Health Information Systems: Study in Seven European Union Countries
Source: JMIR Med Inform. 2018 Nov 30;6(4):e11252. doi: 10.2196/11252 (PMC6294876; doi:10.2196/11252)
Supplement: Multimedia Appendix 6 [file medinform_v6i4e11252_app6.pdf]

Appendix F: Acronyms

ACSI

American Customer Satisfaction Index

AOE

Angular Order of the Eigenvectors

AVE

Average Variance Extracted

CSI

Customer Satisfaction Index

CR

Composite Reliability

COPD

chronic obstructive pulmonary disease

D&M IS

Delone and McLean Information Systems Success

ECSI

European Customer Satisfaction Index

EU

European Union

EUCS

End User Computing Satisfaction

FI-STAR

Future Internet Social and Technological Alignment Research

HOT-fit

Human, Organization, and Technology Fit

HTMT

Heterotrait-Monotrait

LASSO

Least Absolute Shrinkage and Selection Operator

MAST

Model for ASsessment of Telemedicine applications

PLS-SEM

Partial Least Squares Structural Equation Modelling

SRMR

Standardized Root Mean Square Residual

TAM

Technology Acceptance Model

UK

United Kingdom

UTAUT

Unified Theory of Acceptance and Use of Technology

UVON Unified eValuation using Ontology Script
